# Sleep duration promotes resistance and resilience to tau in older women at risk for Alzheimer's disease

**DOI:** 10.1002/alz.71051

**Published:** 2025-12-28

**Authors:** Jordan Stiver, Xin Wang, Kitty K. Lui, Melanie A Dratva, Ella T. Lifset, Nadine C. Heyworth, Charlotte S. Rivera, Atul Malhotra, Michael L. Thomas, Erin E. Sundermann, Sarah J. Banks

**Affiliations:** ^1^ Department of Neurosciences University of California, San Diego La Jolla California USA; ^2^ San Diego Joint Doctoral Program in Clinical Psychology San Diego State University/University of California San Diego California USA; ^3^ Department of Psychology University of California Los Angeles California USA; ^4^ Division of Pulmonary, Critical Care, Sleep Medicine & Physiology, Department of Medicine University of California, San Diego La Jolla California USA; ^5^ Department of Psychology Colorado State University Fort Collins Colorado USA; ^6^ Department of Psychiatry University of California, San Diego La Jolla California USA

**Keywords:** actigraphy, Alzheimer's disease, APOE ε4, memory, modifiable lifestyle factors, older women, resilience, resistance, sex differences, sleep duration, tau PET

## Abstract

**INTRODUCTION:**

Resistance and resilience are pathways through which modifiable behaviors may reduce Alzheimer's disease (AD) risk. Sleep – a known modifiable factor – is understudied in this context, especially among older women at elevated risk for AD.

**METHODS:**

Forty‐five functionally intact older women (≥65 years) at heightened risk for AD completed wrist actigraphy to capture average nocturnal sleep duration. Tau positron emission tomography imaging (^18^F‐MK6240) quantified tau burden across Braak stage regions. Memory was assessed via a delayed recall task composite. Hierarchical regressions tested whether sleep duration moderated the relationship between apolipoprotein epsilon 4 (*APOE* ε4) status and tau (resistance) and between tau and memory (resilience).

**RESULTS:**

Shorter sleep duration amplified the association between *APOE* ε4 status and tau, while longer sleep mitigated it. Similarly, tau burden was related to worse memory performance only among those with short sleep duration.

**DISCUSSION:**

Longer sleep duration may promote resistance and resilience to AD in at‐risk older women, highlighting sleep as a critical intervention target.

**Highlights:**

- Sleep was measured via wrist actigraphy, tau via PET imaging, and memory with a composite score.
- Longer sleep attenuated the link between *APOE* ε4 carriership and tau PET across Braak regions.
- Greater sleep duration weakened the negative impact of tau on memory performance.
- This is the first study to examine sleep in AD resistance and resilience among older women at heightened risk.

## 1 BACKGROUND

The dual framework of “resistance” and “resilience” to Alzheimer's disease (AD) provides a useful lens for understanding how lifestyle factors (e.g., sleep, physical activity, diet) modify disease trajectories. Resistance reflects the capacity to avoid or decelerate AD neuropathogenesis despite risk factors (e.g., genetic vulnerability), whereas resilience reflects preserved cognitive function despite existing AD pathology.^1^, ^2^, ^3^, ^4^ In essence, resistance acts upstream to reduce or delay neuropathogenesis, while resilience operates downstream to buffer its clinical impact.

Lifestyle factors (e.g., healthy sleep) promoting resistance may attenuate the influence of apolipoprotein epsilon 4 (*APOE* ε4) carriership – the strongest genetic risk factor for late‐onset AD.^5^ Proposed mechanisms include efficient protein and metabolic waste clearance^6^ and reduced blood–brain barrier permeability,^7^, ^8^ toxin exposure,^9^, ^10^ neuroinflammation,^11^, ^12^ and oxidative stress.^13^, ^14^ In rodents, sleep results in a two‐fold increase in convective flow between cerebrospinal fluid and interstitial fluid compared to wakefulness, thereby increasing the rate of amyloid beta (Aβ) clearance.^15^, ^16^ Behaviors supporting resilience, in contrast, attenuate the negative impact of AD neuropathology on cognition through growth factor synthesis,^17^, ^18^, ^19^ stronger synaptic and axonal integrity,^20^, ^21^ activation of compensatory neural networks,^22^, ^23^ and increased glucose metabolism.^24^, ^25^ Experimental and neuroimaging evidence suggests that sleep regulates synaptic strength and plasticity,^26^ strengthens network‐level memory consolidation,^27^ and increases cortical metabolic activity,^28^ thereby conferring resilience.

Women are nearly twice as likely as men to develop AD.^29^ Beyond differences in longevity, several female‐specific factors contribute to this disparity, including hypertensive disorders of pregnancy,^30^, ^31^, ^32^, ^33^ menopause‐related changes in brain structure and function,^34^ and epigenetic changes and instability of the X chromosome.^35^ Tau pathology in particular appears central to women's disproportionate risk. Unlike Aβ, which is necessary but insufficient for clinical progression, tau burden shows closer spatial and temporal correspondence with neurodegeneration and cognitive decline.^36^, ^37^, ^38^ Importantly, older women show greater tau accumulation in AD‐specific brain regions – both at preclinical and symptomatic stages of disease, and after accounting for Aβ levels – suggesting sex‐specific vulnerability to tau‐related neurodegeneration.^39^, ^40^ *APOE* ε4 carriership also demonstrates a stronger association with tau signal on positron emission tomography (PET) and in the cerebrospinal fluid of women compared to men, compounding this disparity in tau accumulation and rates of AD.^41^, ^42^, ^43^, ^44^ Finally, lifestyle risk factors (e.g., sedentary behavior) may confer greater AD risk in women.^45^, ^46^, ^47^

Sleep is a particularly relevant, modifiable risk factor among older women, who experience higher rates of insomnia after menopause compared to their male peers.^48^, ^49^, ^50^ The majority of older women obtain fewer than 7 h of sleep per night, as is recommended by the National Sleep Foundation, among other major health organizations, for optimal health outcomes.^51^, ^52^ Numerous studies in cognitively impaired and unimpaired older adults demonstrate a bidirectional relationship between sleep health and AD biomarkers, as well as memory decline – the hallmark clinical manifestation of AD.^53^, ^54^ However, few have examined sleep through the dual lens of resistance and resilience.

Consistent with the resistance pathway, actigraphic markers of sleep quality have moderated the relationship between *APOE* ε4 status and both Aβ PET^55^ and *post mortem* neurofibrillary tangle density,^56^ with higher‐quality sleep attenuating genetic risk effects. Supporting the resilience pathway, Wilckens and colleagues^57^ showed that longer actigraphic sleep duration and higher‐quality sleep moderated the negative effect of Aβ PET on memory. Similarly, a recent polysomnography study showed that greater slow wave activity during sleep buffered the association between Aβ PET positivity and memory.^58^

To our knowledge, no study has examined whether sleep supports both resistance and resilience specifically among older women – a population at elevated risk for AD and heightened sensitivity to lifestyle risk factors. The purpose of this study was to determine whether objective sleep duration promoted resistance and resilience to tau deposition in older women at risk for AD. Consistent with the resistance pathway, we hypothesized that longer sleep duration would attenuate the relationship between *APOE* ε4 status and greater tau PET signal in AD‐related brain regions. Consistent with the resilience pathway, we expected that longer sleep duration would attenuate the relationship between higher tau PET signal and worse memory performance.

**RESEARCH IN CONTEXT**

1. **Systematic review**: Prior studies independently showed that sleep moderated the relationship between genetic risk factors (e.g., *APOE* ε4) and AD biomarkers or between AD biomarkers and cognition. However, no research has examined sleep within the dual framework of AD resistance and resilience, particularly among older women, who face disproportionately higher AD risk and sleep disturbance.
2. **Interpretation**: Longer nocturnal sleep duration may promote both resistance (i.e., by weakening the *APOE* ε4–tau association) and resilience (i.e., by reducing the negative impact of tau pathology on memory) in older women, suggesting that sleep duration is a key modifiable factor in delaying or mitigating AD pathogenesis.
3. **Future directions**: Longitudinal and interventional studies are needed to test causality and assess whether improving sleep duration in at‐risk women slows tau accumulation and cognitive decline. Investigating additional sleep parameters and biological mechanisms underlying sex‐specific vulnerability is also warranted.

## 2 METHODS

### 2.1 Participants and design

Functionally intact older women (*N* = 45) ages 65 and over (*M*_age_ = 72.9, *SD* = 4.3, range: 65.9 to 81.9) were included in this study as part of their participation in the Women: Inflammation and Tau Study (PIs: Sarah J. Banks, Erin E. Sundermann), an ongoing longitudinal cohort study at the University of California, San Diego (UCSD) designed to recruit older women at elevated risk for AD. Participants in the present study represent the initial cohort. Detailed demographic characteristics are presented in Table 1. Study screening included administration of the telephone‐based Montreal Cognitive Assessment (T‐MoCA)^59^ and genotyping of saliva samples by Diagnomics, Inc. (San Diego, CA, USA) using Infinium Global Screening Array versions 2.2 and 3.0. All participants identified as female based on self‐reported sex assigned at birth, were fluent in English and were considered at higher risk for AD based on a score in the range of 13 to 20 on the T‐MoCA and either having an AD polygenic hazard score (PHS) of ≥50%^60^ and/or a self‐reported family history of dementia. Exclusionary criteria included a formal diagnosis of dementia and/or neurodegenerative diseases, major medical illness, serious psychiatric illness, active substance dependence, active participation in an investigational drug study, magnetic resonance imaging (MRI) contraindications, and select medications including central nervous system agents (e.g., neuroleptics, anticonvulsants) and potent anti‐inflammatory medications (e.g., hydrocortisone, methotrexate).

**TABLE 1:** Demographic and study variable descriptive statistics (*N* = 45).

	Overall sample (*N *= 45)			*APOE* ε4 negative (*n *= 23)			*APOE* ε4 positive (*n *= 22)			
Variable	*M* or *n*	SD or %	Range	*M* or *n*	SD or %	Range	*M* or *n*	SD or %	Range	*t* or χ^2^
Age	72.9	4.3	65.9 to 81.9	72.9	4.3	65.9 to 80.9	72.9	4.4	66.1 to 81.9	0.0
Education	16.2	1.8	11 to 20	16.3	1.7	12 to 20	16.1	1.9	11 to 18	0.2
**Race/Ethnicity**										0.7^a^
Latinx (any race)	3	6.7%	—	2	8.7%	—	1	4.5%	—	
Non‐Latinx Asian	2	4.4%	—	2	8.7%	—	0	0.0%	—	
Non‐Latinx White	39	86.7%	—	19	82.6%	—	20	90.9%	—	
Non‐Latinx, two or more races	1	2.2%	—	0	0.0%	—	1	4.5%	—	
PHS	65.4	25.0	3 to 99	47.4	21.9	3 to 70	84.2	8.9	72 to 99	−7.3^***^
***APOE* genotype**										
ε2/ε2	0	0.0%	—	0	0.0%	—	0	0.0%	—	
ε2/ε3	2	4.4%	—	2	8.7%	—	0	0.0%	—	
ε2/ε4	1	2.2%	—	0	0.0%	—	1	4.5%	—	
ε3/ε3	21	46.7%	—	21	91.3%	—	0	0.0%	—	
ε3/ε4	18	40.0%	—	0	0.0%	—	18	81.8%	—	
ε4/ε4	3	6.7%	—	0	0.0%	—	3	13.6%	—	
**Actigraphy**										
No. of valid nights	7.4	1.4	4 to 13	7.2	1.2	4 to 10	7.7	1.5	5 to 13	−1.1
TST, min	424.7	59.9	265.6 to 556.3	416.3	71.1	265.6 to 556.3	433.6	45.3	323.3 to 433.6	−1.0
Obtained ≥7 h TST	22	48.9%	—	10	43.5%	—	12	54.5%	—	0.6^b^
Nappers	39	86.7%	—	19	82.6%	—	20	90.9%	—	0.7^c^
Naps/week	3.3	2.5	0.0 to 9.3	3.5	2.7	0.0 to 9.3	3.1	2.6	0.0 to 9.0	0.6
**Cognitive status**										3.0
Cognitively unimpaired	32	71.1%	—	19	82.6%	—	13	59.1%	—	
Mild cognitive impairment	13	28.9%	—	4	17.4%	—	9	40.9%	—	
**Cognitive outcomes**										
T‐MoCA	18.6	1.7	16 to 22	18.5	1.7	16 to 22	18.8	1.688	16 to 22	−0.6
WRAT4‐WR, raw score	64.5	3.9	55 to 70	63.6	4.009	55 to 70	65.5	3.7	58 to 70	−1.6
WRAT4‐WR, standard score	114.0	14.7	91 to 145	110.9	13.3	91 to 145	117.2	15.8	95 to 145	−1.5
WMS‐R LM Delayed Recall, raw score	20.6	6.8	6 to 35	18.8	5.0	10 to 28	22.5	7.9	6 to 35	−1.9
WMS‐R LM Delayed Recall, z‐score	0.0	1.0	−2.2 to 2.1	−0.3	0.7	−1.6 to 1.1	0.3	1.2	−2.2 to 2.1	−1.9
RAVLT Delayed Recall, raw score	8.3	3.5	0 to 14	8.3	2.7	0 to 13	8.3	4.2	0 to 14	0.0
RAVLT Delayed Recall, z‐score	0.0	1.0	−2.4 to 1.6	0.0	0.8	−2.4 to 1.4	0.0	1.2	−2.4 to 1.6	0.0
BVMT‐R Delayed Recall, raw score	8.5	2.8	0 to 12	9.0	2.1	4 to 12	7.9	3.3	0 to 12	1.3
BVMT‐R Delayed Recall, z‐score	0.0	1.0	−3.0 to 1.3	0.2	0.7	−1.6 to 1.3	−0.2	1.2	−3.0 to 1.3	1.3
Memory composite	0.0	0.7	−2.0 to 1.4	0.0	0.5	−1.2 to 0.9	0.0	0.9	−2.0 to 1.4	−0.2
**^18^F‐MK6240 Tau PET**										
Tau PET positive	10	22.2%	—	2	8.7%	—	8	36.4%	—	5.0^*^
Braak I/II	1.02	0.27	0.68 to 1.81	0.94	0.15	0.68 to 1.27	1.12	0.33	0.69 to 1.81	−2.4^*^
Braak III/IV	1.05	0.17	0.79 to 1.82	1.00	0.11	0.79 to 1.33	1.10	0.20	0.82 to 1.82	−2.1^*^
Braak V/VI	0.96	0.10	0.72 to 1.15	0.93	0.10	0.72 to 1.10	0.98	0.10	0.77 to 1.15	−1.8

Abbreviations: *APOE* ε4, apolipoprotein E epsilon 4; PHS, polygenic hazard score; TST, total sleep time; T‐MoCA, telephone‐based Montreal Cognitive Assessment; WRAT4‐WR, Wide Range Achievement Test, Fourth Edition, Word Reading; WMS‐R LM, Wechsler Memory Scale–Revised, Logical Memory; RAVLT, Rey Auditory Verbal Learning Test; BVMT‐R, Brief Visuospatial Memory Test–Revised; PET, positron emission tomography.

^a^ Non‐Latinx White = 0, any other race/ethnicity = 1.

^b^ Obtained < 7 h TST = 0, obtained ≥7 h TST = 1.

^c^ Took 0 naps = 0, Took ≥1 naps = 1.

* *p* < 0.05,

*** *p* < 0.001.

This study required three visits to the laboratory. During the first visit, participants were provided an actigraphic monitoring device (Actiwatch Spectrum PRO from Philips Respironics, Inc.) to wear continuously on the non‐dominant wrist for up to 2 weeks. During this period, participants also completed a daily sleep diary to record their sleep habits (e.g., time they went to bed, time they fell asleep, time of final awakening).^61^ Following the sleep study period, participants returned for a second study visit. During the second visit, participants returned the Actiwatch for data download, completed a cognitive test battery, and underwent brain MRI. Participants then returned for a third visit to complete tau PET. All study visits for each participant were completed within a month. All participants provided informed consent and were compensated financially for their involvement. The UCSD's Institutional Review Board approved the study protocol.

### 2.2 Actigraphy

Actigraphic data were recorded for each participant in 30‐s epochs via the Actiwatch Spectrum PRO (Philips Respironics) worn continuously on the non‐dominant wrist. Actigraphic data were extracted using Actiware version 6.3.0 (Philips Respironics). Individual epochs were scored as either sleep or wake based on the default, medium sensitivity activity count threshold (i.e., 40) preset of the Actiware scoring algorithm. At this preset, sleep onset and offset were defined as 10 consecutive minutes scored as sleep and 10 consecutive minutes scored as awake, respectively – an approach to detecting sleep onset shown to be reliable and valid as compared to polysomnography.^62^ Congruence between actigraphy‐ and sleep diary‐derived measures of sleep and wake timing was visually inspected by a trained member of the research team, and any resultant discrepancies were corrected as needed in the actigraphy output. The reliability of rest intervals was determined by the concordance of (1) changes in activity level, (2) changes in light exposure, and (3) self‐report via the sleep diary. When these three factors did not agree (e.g., when activity counts dropped to zero while ambient light remained bright, or when high activity persisted in darkness late in the night), the sleep diary was used to assist in determining rest interval start and end times. This approach was used to minimize measurement error and correct for behaviors such as pre‐sleep electronics usage that actigraphic devices occasionally identify as restlessness while attempting to sleep or even sleep itself.

Sleep duration over each night for each participant was defined as the total sleep time (TST) (i.e., total time scored as sleep, in minutes) between sleep onset and sleep offset. Mean TST was calculated for each participant's sleep study period. A minimum of three consecutive nights of actigraphic data was required for inclusion in analyses based on recommendations by Aili et al.^63^ for obtaining reliable mean estimates of objective TST. Any isolated rest intervals that were separate from participants’ primary nocturnal rest intervals were coded as naps according to the same procedures described above. Frequency of daytime napping (i.e., naps per week) was included as a covariate to isolate the effects of nocturnal sleep, as napping may reflect a compensatory behavior for suboptimal nighttime sleep and is not equivalent to nocturnal restorative processes.^64^, ^65^

### 2.3 Cognition

Participants completed a comprehensive neuropsychological test battery and were classified as cognitively unimpaired or as having mild cognitive impairment based on the Jak/Bondi actuarial neuropsychological criteria.^66^ For the current analyses, we were particularly interested in memory performance given its relevance in AD, and we used a proxy measure of premorbid intellectual functioning and quality of education (i.e., the total raw score of the Word Reading subtest from the Wide Range Achievement Test, Fourth Edition [WRAT4‐WR])^67^, ^68^ as a covariate. Specifically, the WRAT‐WR better captures educational quality compared to years of education, which does not account for the variability in quality of education that is related to sociocultural factors and/or structural inequities.^23^, ^69^

We used three memory tests. The logical memory subtest from the Wechsler Memory Scale–Revised (WMS‐R LM)^70^ and the Rey Auditory Verbal Learning Test (RAVLT)^71^ were used to measure verbal episodic memory; non‐verbal memory was assessed using Form 2 from the Brief Visuospatial Memory Test–Revised (BVMT‐R).^72^ Raw scores from the delayed recall trials of each test were converted to z‐scores using the sample's means and standard deviations; the three resultant z‐scores were then averaged for each participant to compute a composite memory score for analyses.

### 2.4 Neuroimaging

#### 2.4.1 Brain MRI

MRI scans were acquired on a 3.0T GE 750 scanner (i.e., following the Alzheimer's Disease Neuroimaging Initiative MRI protocol)^73^ IR‐FSPGR (TI = 400 ms, FA = 11°) at the UCSD Altman Clinical Translational Research Institute. T1‐weighted scanned images were processed with FreeSurfer (version 7.1.1) to derive volumetric measures for regions of interest (ROIs) using the Desikan‐Killiany Atlas. Visual inspection of segmented regions and manual edits were completed by trained staff.

#### 2.4.2 TAU PET imaging

PET with ^18^F‐MK6240 to assess tau deposition was done on a GE Discovery 610 PET/CT scanner at California Protons Cancer Therapy Center (San Diego, CA, USA). Participants were injected with an ^18^F‐MK6240 tracer dose of 5.0 mCi ± 10% (5.0 mCi = 185 MBq). Image acquisition began 80 min after injection, with six 5‐min frames collected over a total of 30 min in the scanner. Smoothing, interframe realignment, and co‐registration of four‐dimensional tau PET to T1‐weighted MRI were performed using SPM12. The first four frames (i.e., starting at 80 min after injection) were then summed to generate standard uptake value ratios (SUVRs) with the eroded inferior cerebellum gray matter as the reference region. The eroded inferior cerebellum was created from the Automated Anatomical Labeling atlas, and tau PET processing adhered to methods described by Betthauser and colleagues.^74^ Because tau deposition follows a predictable temporal pattern of Braak staging,^75^ tau PET volume‐weighted composite SUVRs were calculated in Braak stage groupings I/II (i.e., entorhinal cortex and hippocampus), III/IV (i.e., limbic regions), and V/VI (i.e., neocortex),^37^, ^75^ as well as a temporal meta‐ROI comprising the amygdala, entorhinal cortices, fusiform gyri, and inferior and medial temporal cortices to determine rates of tau PET positivity (i.e., at a SUVR threshold of 1.24).^76^

### 2.5 Statistical analyses

All data were analyzed using IBM SPSS Statistics for Macintosh version 30.0 (IBM Corp., Armonk, NY, USA). To determine whether sleep duration moderated the effect of *APOE* ε4 carrier status on tau deposition, we ran a series of three hierarchical regression models predicting tau PET signal composites from each Braak stage grouping. Independent variables included *APOE* ε4 carrier status, TST, and two covariates (i.e., age and naps per week) entered at step 1 and an *APOE* ε4 carrier status by TST interaction term entered at step 2. *APOE* ε4 carrier status and TST were mean‐centered prior to analyses and computation of their interaction term to reduce multicollinearity and facilitate interpretation of interaction effects.^77^ To assess the moderating effect of sleep duration on the relationship between tau deposition and memory function, we ran another series of three hierarchical regression models predicting memory composite score performance. Independent variables included tau PET signal (i.e., at each Braak stage grouping per model), TST, and four covariates (i.e., age, naps per week, *APOE* ε4 carrier status, and WRAT4‐WR) entered at step 1 and a tau PET signal (i.e., at each Braak stage grouping per model) by TST interaction term entered at step 2. Tau PET variables and TST were mean‐centered prior to analyses and computation of their interaction terms. Interactions were probed via analyses of simple slopes. For each interaction model, this procedure involved calculating the conditional effects of the independent variable on the dependent variable at values of −1 SD (i.e., “low”) and +1 SD (i.e., “high”) of the moderator (i.e., TST).^77^, ^78^

For all regression models, we examined potential influential cases using standard diagnostic metrics including Cook's distance, leverage (hat values), studentized residuals, and differences in beta, scaled (DFBETAS). Thresholds for identifying potentially influential observations were based on conventional guidelines (i.e., Cook's distance > 4/*N*; leverage > 2(k+1)/*N*; |studentized residual| > 3; |DFBETAS| > 2/√*N*). Only observations flagged on more than one diagnostic per interaction model were considered for sensitivity analyses. Corresponding interaction models were subsequently re‐estimated excluding these participants to confirm the primary results were not driven by individual observations.

## 3 RESULTS

Sample demographics and descriptive statistics for all study variables of interest are presented in Table 1. Briefly, the overall sample had a mean age of 72.9 years (SD = 4.3; range = 65.9 to 81.9) and a mean education level of 16.2 years (SD = 1.8; range = 11 to 20). The majority of the sample (86.7%; *n *= 39) identified as non‐Latinx White; 6.7% (*n *= 3) identified as Latinx (any race), 4.4% (*n *= 2) identified as non‐Latinx Asian, and 2.2% (*n *= 1) identified as non‐Latinx, two or more races. As a result of our inclusion/exclusion criteria that were intended to yield a sample of older women enriched for elevated AD risk, approximately half of the sample (48.9%; *n *= 22) carried at least one *APOE* ε4 allele. Just over a quarter of the sample (28.9%; *n *= 13) was classified as having mild cognitive impairment, and 22.2% (*n *= 10) were positive for tau PET. *APOE* ε4 carriers had a higher PHS (*p* < 0.001) as well as a higher rate of tau PET positivity and greater tau PET signal in Braak I/II and III/IV regions (*p*s < 0.05) compared to non‐carriers. There were no significant group differences in any demographic or other study variables of interest by *APOE* ε4 carrier status (*p*s > 0.05).

**FIGURE 1:**
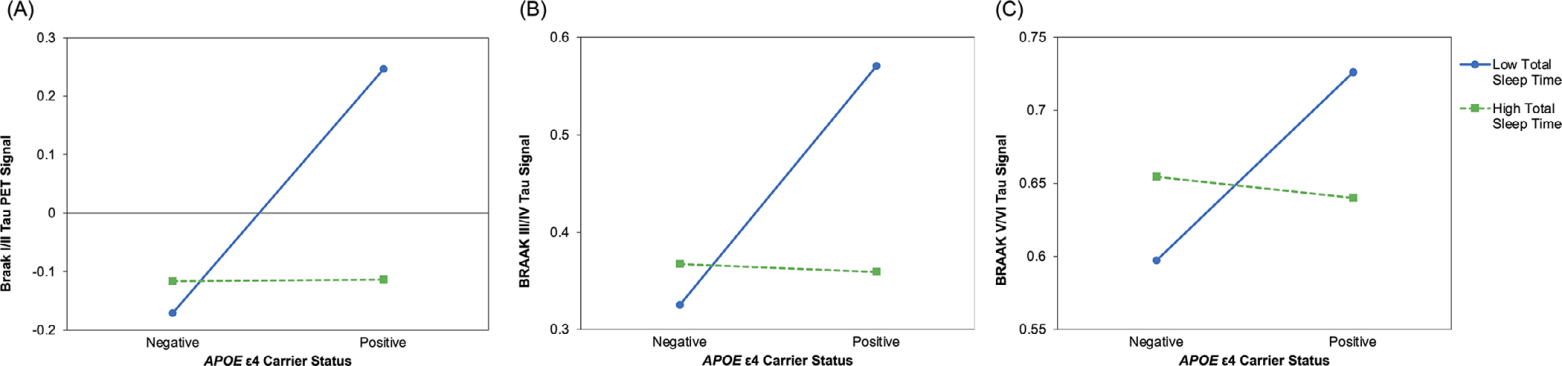
Plots showing interactive effects of *APOE* ε4 and total sleep time on tau PET signal at Braak stage grouping I/II (A), III/IV (B), and V/VI (C). Predictors are mean‐centered, and covariates are fixed at their sample means.*APOE* ε4,  apolipoprotein E epsilon 4; PET, positron emission tomography.

There were significant *APOE* ε4 carrier status by TST interactions on tau PET signal at each Braak stage grouping (Table 2). Specifically, *APOE* ε4 carrier status interacted with TST to predict tau PET signal in Braak I/II (*β* = −0.37, *p* = 0.020), Braak III/IV (*β* = −0.36, *p* = 0.027), and Braak V/VI (*β* = −0.35, *p* = 0.035) regions. Consistent with the significant interaction effects, simple slopes analyses revealed that at the specified low level of TST, *APOE* ε4 positivity was associated with greater tau PET signal at each Braak stage grouping (Figure 1), i.e., at Braak I/II (*β* = 0.77, *p* = 0.001), Braak III/IV (*β* = 0.72, *p* = 0.003), and Braak V/VI (*β* = 0.65, *p* = 0.007). At high TST, there were no significant relationships between *APOE* ε4 status and tau PET signal at any Braak stage grouping (*p*s > 0.05).

**TABLE 2:** Hierarchical regression models showing interactive effects of *APOE* ε4 and total sleep time on tau PET signal by Braak stage grouping.

Models	*B*	*SE B*	β	*t*	*p*	*R^2^*	*p*(*R* ^2^)
**Model 1 DV: Braak I/II**							
Step 1	—	—	—	—	—	0.17	0.104
*APOE* ε4 status	0.193	0.078	0.36	2.46	0.018	—	—
TST	−0.001	0.001	−0.13	−0.87	0.391	—	—
Step 2	—	—	—	—	—	0.28	0.021
Interaction: *APOE* ε4 status × TST	−0.003	0.001	−0.37	−2.43	0.020	—	—
**Model 2 DV: Braak III/IV**							
Step 1	—	—	—	—	—	0.14	0.193
*APOE* ε4 status	0.109	0.050	.32	2.16	0.037	—	—
TST	−0.0002	0.0004	−0.10	−0.66	0.513	—	—
Step 2	—	—	—	—	—	0.24	0.048
Interaction: *APOE* ε4 status × TST	−0.002	0.001	−0.36	−2.31	0.027	—	—
**Model 3 DV: Braak V/VI**							
Step 1	—	—	—	—	—	0.10	0.360
*APOE* ε4 status	0.051	0.030	0.26	1.73	0.092	—	—
TST	0.0001	0.0002	0.07	0.49	0.626	—	—
Step 2	—	—	—	—	—	0.20	0.111
Interaction: *APOE* ε4 status × TST	−0.001	0.001	−0.35	−2.18	0.035	—	—

*Note*: Predictors were mean‐centered prior to analysis. Models are adjusted for age and naps per week.

Abbreviations: *APOE* ε4, apolipoprotein E epsilon 4; PET, positron emission tomography; DV, dependent variable; TST, total sleep time.

**FIGURE 2:**
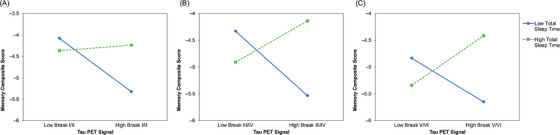
Plots showing interactive effects of Braak stage grouping‐specific tau positron emission tomography signal, i.e., Braak stage grouping I/II (A), III/IV (B), and V/VI (C), and total sleep time on memory performance. Predictors are mean‐centered and covariates are fixed at their sample means.

There were significant interactions between tau PET signal at each Braak stage grouping and TST on memory performance (Table 3). Specifically, TST interacted with tau PET signal in Braak I/II (*β* = 0.31, *p* = 0.043), Braak III/IV (*β* = 0.42, *p* = 0.031), and Braak V/VI (*β* = 0.46, *p* = 0.009) regions to predict memory performance. Simple slope analyses indicated that tau PET signal at each Braak stage grouping was associated with worse memory composite score performance at low TST (Figure 2), i.e., Braak I/II (*β* = −0.85, *p* < 0.001), Braak III/IV (*β* = −0.83, *p* = 0.002), and Braak V/VI (*β* = −0.56, *p* = 0.013). At high TST, there were no significant relationships between Braak I/II or III/IV tau PET signal and memory composite score performance (*p*s > 0.05); however, Braak V/VI tau PET signal was marginally positively associated with memory composite score performance (*β* = 0.64, *p* = 0.049).

**TABLE 3:** Hierarchical regression models showing interactive effects of Braak stage grouping‐specific tau PET signal and total sleep time on memory performance.

	DV: Memory composite						
Models	*B*	*SE B*	*β*	*t*	*p*	*R^2^*	*p*(*R* ^2^)
**Model 1**							
Step 1	—	—	—	—	—	0.33	0.014
Braak I/II	−1.202	0.392	−0.45	−3.07	0.004	—	—
TST	0.002	0.002	0.20	1.44	0.159	—	—
Step 2	—	—	—	—	—	0.40	0.005
Interaction: Braak I/II × TST	0.021	0.010	0.31	2.10	0.043	—	—
**Model 2**							
Step 1	—	—	—	—	—	0.29	0.035
Braak III/IV	−1.623	0.629	−0.38	−2.58	0.014	—	—
TST	0.003	0.002	0.22	1.53	0.134	—	—
Step 2	—	—	—	—	—	0.37	0.010
Interaction: Braak III/IV × TST	0.048	0.021	0.42	2.25	0.031	—	—
**Model 3**							
Step 1	—	—	—	—	—	.18	.257
Braak V/VI	−0.908	1.156	−0.12	−0.79	0.437	—	—
TST	0.003	0.002	0.26	1.73	0.093	—	—
Step 2	—	—	—	—	—	0.32	0.037
Interaction: Braak V/VI × TST	0.074	0.027	0.46	2.75	0.009	—	—

*Note*: Predictors were mean‐centered prior to analysis. Models are adjusted for age, naps per week, *APOE* ε4 status, and the Word Reading subtest from the Wide Range Achievement Test, Fourth Edition.

Abbreviations: DV, dependent variable; PET, positron emission tomography; TST, total sleep time.

Diagnostic screening for influential cases identified one participant each in Model 2 from the resistance analyses presented in Table 2 and Model 1 from the resilience analyses presented in Table 3 who exceeded more than one conservative threshold (i.e., Cook's distance and leverage or studentized residual). Re‐estimating these models without the flagged participants (i.e., *n* = 44) yielded nearly identical results: The focal interaction terms remained statistically significant (*p*s < 0.05), and in both cases, the effects were slightly stronger in magnitude (Model 2 from resistance analyses interaction term *β* = −0.36 → ‐0.37; Model 1 from resilience analyses interaction term *β* = 0.31 → 0.34), suggesting the observed interactions are not driven by single influential data points. To facilitate greater visualization of underlying data distributions and interpretation of interaction effects, Figures S1‐S4 present corresponding boxplots and scatterplots with individual unadjusted, raw data observations as well as unstandardized, regression model‐predicted values.

## 4 DISCUSSION

This study provides compelling evidence that nocturnal sleep duration may serve as a key modifiable lifestyle factor in promoting both resistance and resilience to AD among older women at elevated risk for AD. Specifically, among those with shorter average sleep duration, *APOE* ε4 positivity was related to greater tau deposition across all Braak stage groupings, whereas longer sleep duration completely attenuated this relationship. These findings support the hypothesis that sufficient nighttime sleep may help mitigate the neuropathological consequences of genetic risk, consistent with the resistance pathway. Similarly, we observed that a greater amount of sleep mitigated the negative effects of tau pathology on memory function, consistent with the resilience pathway. Interestingly, in late‐stage Braak regions (i.e., V/VI), longer sleep duration was not only protective but even appeared to reverse the expected association between tau and memory – higher tau signal was unexpectedly associated with better memory performance among longer sleepers. While this latter finding may reflect a spurious association, it is also possible that those individuals who can maintain longer sleep durations in the face of greater late‐stage tau deposition are also more likely to have adhered to other unmeasured aspects of a healthy lifestyle and have fewer comorbidities, thereby making them extraordinarily resilient to the effects of more significant AD pathology.^79^, ^80^

The current findings extend prior work in several important ways. While previous individual studies demonstrated that sleep either moderates the effect of *APOE* ε4 status on AD biomarkers or of AD biomarkers on cognition, none approached these relationships within the dual framework of resistance and resilience. In addition, no study has examined these relationships specifically in women, despite evidence suggesting that older women face a disproportionately higher burden of both sleep disturbance and AD risk.^29^, ^48^, ^49^, ^50^ Our findings suggest that sleep duration may serve as an essential modifiable risk factor, particularly in the context of *APOE* ε4 carriership – a well‐established genetic risk factor that appears to exert a greater negative impact on women.^41^, ^42^, ^43^, ^44^ Importantly, we utilized a rigorous, objective measurement of sleep duration using wrist actigraphy (i.e., as opposed to relying on self‐report), we adjusted for daytime napping, and our results held across stages of tau progression in AD (i.e., Braak stage groupings), lending robustness to the observed moderating effects of nocturnal sleep duration.

Several additional strengths of the study, as well as a number of limitations, are worth highlighting. The use of the tau PET tracer ^18^F‐MK6240 allowed for sensitive and specific detection of tau pathology in vivo, including in early Braak stages relevant to preclinical AD. Participants were also well characterized; the WITS applied strict inclusion and exclusion criteria to yield an enriched cohort of older women at risk for AD while minimizing the confounding effects of other neurological, psychiatric, and medical comorbidities. Finally, the use of a composite memory outcome based on both verbal and non‐verbal measures that were administered soon after participants’ sleep study period enabled us to capture a broad and reliable index of memory function that was close in temporal proximity to our objective sleep metric. While the current sample size (*N* = 45) was modest, it was comparable to that of prior biomarker studies given the resource‐intensive nature of combining multiple neuroimaging techniques and objective sleep measurement. The sample was also relatively homogeneous (e.g., 86.7% non‐Latinx white, ≥11 years of education), thereby limiting the generalizability of our results to older women of underrepresented ethnocultural backgrounds and/or from more diverse educational backgrounds. Our sample's range of sleep duration was also limited to approximately 3 to 9 h per night, suggesting there were no “long sleepers,” commonly defined as those obtaining more than 9 to 10 h per night. Accordingly, although this study was guided by well‐established mechanistic hypotheses linking sleep, AD pathology, and memory function, the relatively small sample warrants cautious interpretation and underscores the importance of replication in larger, more representative cohorts. While we attempted to control for potential confounders beyond age (e.g., daytime napping, educational quality), other unexplored variables such as hormonal status, undiagnosed sleep disorders (e.g., obstructive sleep apnea), mood symptoms, pain, and/or other lifestyle factors like physical activity and diet may have influenced the observed associations. Furthermore, the cross‐sectional design prevents any causal conclusions, though the observed interactions provide a strong basis for future longitudinal studies. Finally, while actigraphy offers a reliable and ecologically valid estimate of nocturnal sleep duration, it does not capture detailed information about sleep architecture (e.g., slow wave activity), which may pose unique contributions to AD resistance and resilience.

Future longitudinal studies of representative samples will be critical in determining whether a healthy duration of sleep prevents the accumulation of tau pathology and/or slows cognitive decline in at‐risk women over time. It will also be important to test how other aspects of sleep health (e.g., sleep quality, circadian features) interact with AD biomarkers and/or whether specific stages of sleep are particularly impactful. Intervention studies targeting sleep in midlife and/or early older adulthood could provide causal evidence that improving sleep duration or quality reduces AD risk, especially in *APOE* ε4 carriers. Additionally, examining hormonal, endocrine, and metabolic mechanisms linking sleep and tau pathology could shed light on why women may be vulnerable to adverse effects of poor sleep on brain and cognitive aging.

## 5 CONCLUSION

In summary, our findings suggest that sleep duration may be an active promoter of resistance and resilience to AD in the face of genetic and neuropathological risk. In older women, who are disproportionately affected by both AD and sleep disturbance, supporting healthy sleep behaviors may be an effective strategy for delaying or reducing the impact of disease. These insights have important implications for public health and disease prevention, particularly for women navigating the menopausal transition – a critical window during which both sleep and AD risk trajectories are known to shift.

## CONFLICT OF INTEREST STATEMENT

Dr. Malhotra is funded by the NIH. He reports income from Eli Lilly, Livanova, Sunrise, Zoll, and Powell Mansfield. He is co‐founder of Clairyon, a small startup unrelated to this topic. Resmed provided a philanthropic donation to the UCSD. All other authors declare no conflicts of interest.

## CONSENT STATEMENT

The UCSD's Institutional Review Board approved the study protocol, and all participants provided written informed consent.

## Supporting information



Supporting Information

Supporting Information
