# Supplementary material for: Sleep duration promotes resistance and resilience to tau in older women at risk for Alzheimer's disease
Source: Alzheimers Dement. 2025 Dec 28;21(12):e71051. doi: 10.1002/alz.71051 (PMC12745661; doi:10.1002/alz.71051)
Supplement: Supplementary file 2 — Supporting Information [file ALZ-21-e71051-s002.pdf]

FIGURE S1

Boxplots showing interactive effects of *APOE* ε4 and total sleep time (TST; median-split for visualization) on tau PET signal at Braak stage grouping I/II (A), III/IV (B), and V/VI (C).

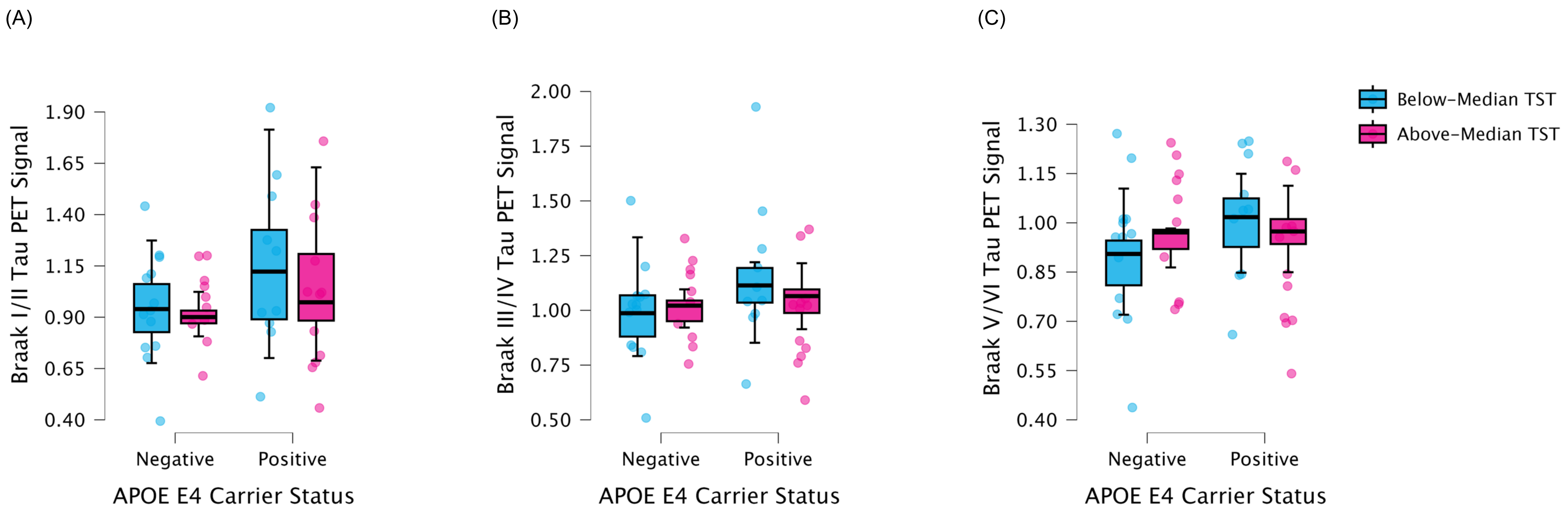

Note: APOE ε4 = apolipoprotein E epsilon 4; PET = positron emission tomography; TST = total sleep time. Values corresponding to y-axes represent unadjusted, raw values.

FIGURE S2

Boxplots showing interactive effects of *APOE* ε4 and total sleep time (TST; median-split for visualization purposes) on tau PET signal at Braak stage grouping I/II (A), III/IV (B), and V/VI (C).

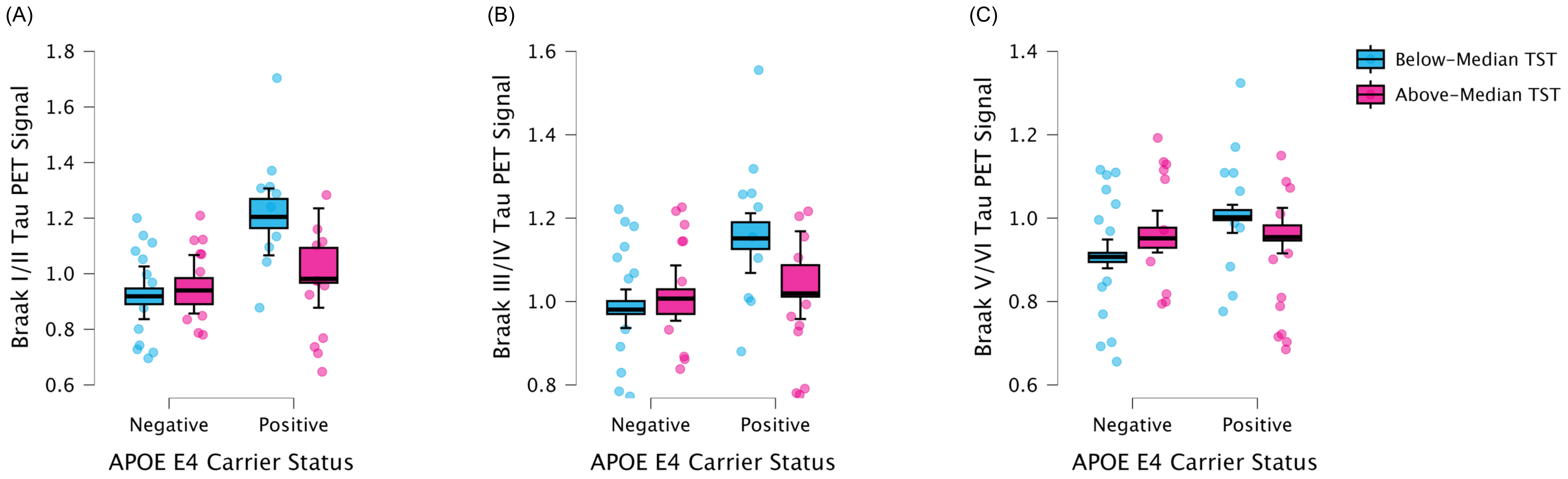

Note: SD = standard deviation; PET = positron emission tomography; TST = total sleep time. Values corresponding to y-axes represent unstandardized predicted values from corresponding regression models.

FIGURE S3

Scatterplots showing interactive effects of Braak stage grouping-specific tau PET signal, i.e., Braak stage grouping I/II (A), III/IV (B), and V/VI (C), and total sleep time (i.e., grouped by < -1 standard deviation, -1 to +1 standard deviation, and > +1 standard deviation for visualization purposes) on memory performance.

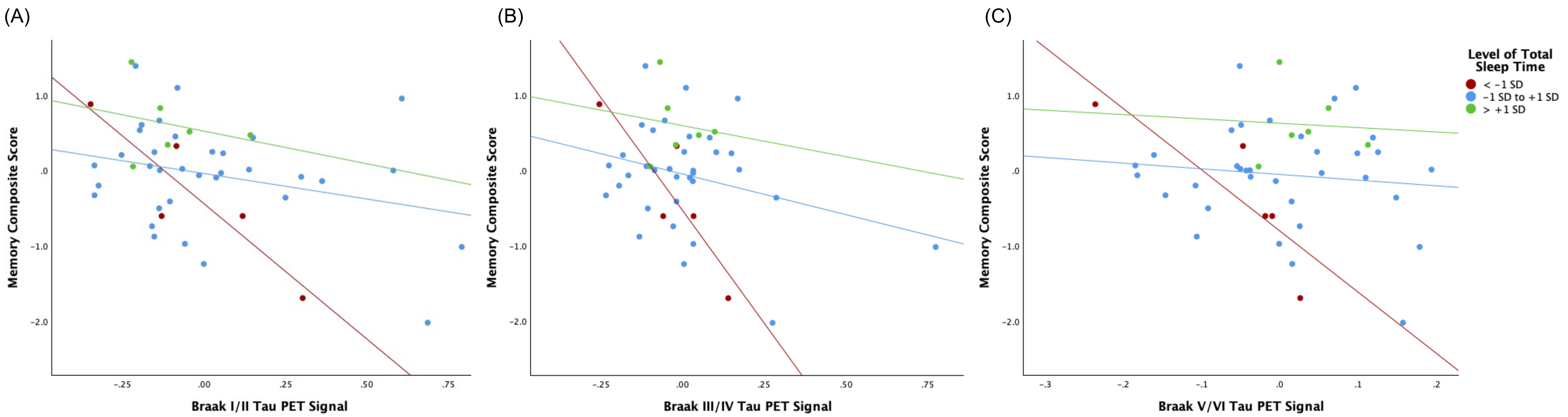

Note: SD = standard deviation; PET = positron emission tomography. Values corresponding to x-axes are mean-centered. Y-axes represent unadjusted, raw values.

FIGURE S4

Scatterplots showing interactive effects of Braak stage grouping-specific tau PET signal, i.e., Braak stage grouping I/II (A), III/IV (B), and V/VI (C), and total sleep time (i.e., grouped by < -1 standard deviation, -1 to +1 standard deviation, and > +1 standard deviation for visualization purposes) on memory performance.

(A) (B) (C)

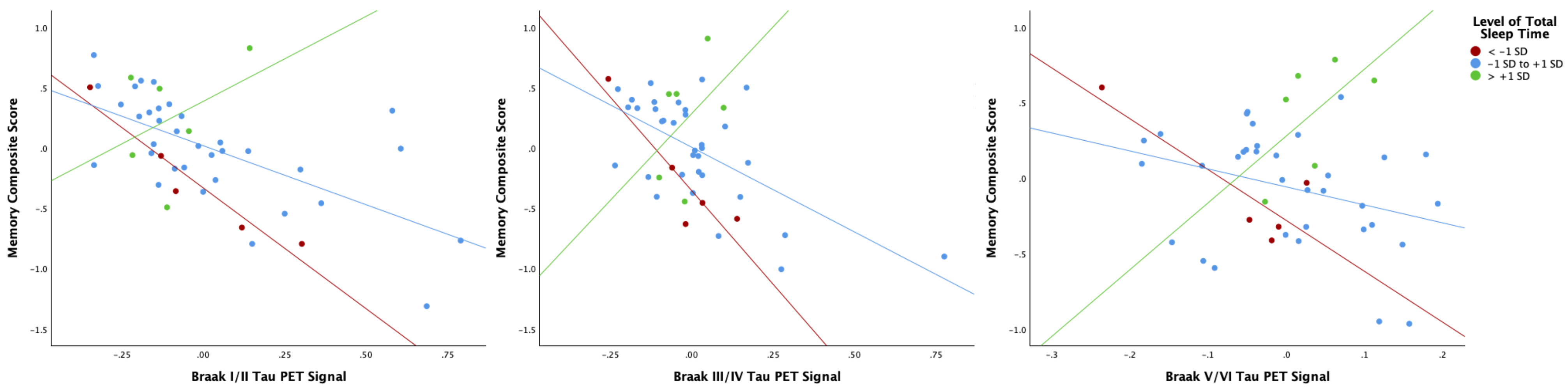

Note: SD = standard deviation; PET = positron emission tomography. Values corresponding to x-axes are mean-centered. Y-axes represent unstandardized predicted values from corresponding regression models.
